# Perils and progress in epilepsy surgery utilization: Twenty‐five years later

**DOI:** 10.1002/epi.70419

**Published:** 2026-08-18

**Authors:** Dario J. Englot

**Affiliations:** ^1^ Department of Neurological Surgery Vanderbilt University Medical Center Nashville Tennessee USA

**Keywords:** drug‐resistant epilepsy, epilepsy surgery, health care disparities, laser interstitial thermal therapy, neuromodulation, referral

## Abstract

More than 25 years have passed since the first randomized controlled trial (RCT) established that surgery is superior to continued anti‐seizure medication (ASM) for drug‐resistant temporal lobe epilepsy, and nearly as long since a joint practice parameter urged that appropriate surgical candidates be referred to a specialized center for evaluation—guidance that subsequent consensus has since broadened to all patients with drug‐resistant epilepsy. Despite Class I evidence and durable guidelines, epilepsy surgery remains one of the most underutilized effective interventions in medicine: fewer than 1% of potentially eligible patients are referred, and those who are referred reach a center on average two decades after seizure onset. This critical review, framed primarily around U.S. data and supplemented by a dedicated assessment of the global picture, examines the scope and burden of drug‐resistant epilepsy (DRE), the foundational evidence and guideline history, and the magnitude, patterns, and causes of persistent underutilization, including a distinct discussion of pediatric care and of disparities by race, insurance, and geography. We then turn to grounds for optimism. Surgical candidacy has materially expanded through minimally invasive approaches to both monitoring (stereo‐electroencephalography) and treatment (laser interstitial thermal therapy and neuromodulation), and indications have broadened to include selected generalized and multifocal epilepsies through thalamic neuromodulation. Contemporary International League Against Epilepsy (ILAE) recommendations go further still, extending referral even to selected patients who are seizure‐free on medication but harbor a surgically accessible lesion. We also raise a counterbalancing concern: the welcome diffusion of minimally invasive options should not eclipse resective and disconnective surgery, which remain the only potentially curative options validated by RCTs. We conclude that, 25 years on, underutilization persists as a major and partly remediable problem, and we offer recommendations for closing the surgical gap.

**Key points**

- Despite Class I evidence and guidelines, <1% of people with drug‐resistant epilepsy are referred for surgery, often after ~20 years.
- Underutilization reflects provider misconceptions, patient fears, referral barriers, and disparities by race, insurance, and geography.
- Seeing surgery as “palliative” deters referral; the International League Against Epilepsy (ILAE) now suggests referral even for some seizure‐free patients with a resectable lesion.
- Minimally invasive monitoring, ablation, and neuromodulation have broadened candidacy, including generalized and multifocal epilepsy.
- Resective/disconnective surgery, the only randomized controlled trial (RCT)–validated potentially curative option, must be preserved as minimally invasive therapies grow.

## 1 INTRODUCTION

In 2001, Wiebe and colleagues reported the first randomized controlled trial (RCT) of epilepsy surgery, demonstrating that, among adults with drug‐resistant temporal lobe epilepsy, anteromesial temporal resection rendered 58% of patients free of seizures that impaired awareness of self and surroundings at 1 year, compared with 8% of those randomized to continued medical therapy.^1^ Two years later, a joint practice parameter from the American Academy of Neurology (AAN), the American Epilepsy Society (AES), and the American Association of Neurological Surgeons (AANS) reviewed this Class I evidence alongside numerous case series and concluded that patients disabled by such seizures should be referred to a tertiary epilepsy center for surgical evaluation.^2^ The threshold for that referral was subsequently operationalized: in 2010 the International League Against Epilepsy (ILAE) defined drug‐resistant epilepsy (DRE) as failure of two tolerated, appropriately chosen and used anti‐seizure medication (ASM) regimens to achieve sustained seizure freedom.^3^

A quarter‐century later, the central paradox of the field is that this evidence has not translated into practice. As Jerome Engel Jr. observed in his Wartenberg Lecture, “surgery for epilepsy remains, arguably, the most underutilized of all acceptable medical interventions, and the reasons for this are unclear.”^4^ Fewer than 1% of patients with DRE are referred for evaluation, and those who do reach a comprehensive center arrive, on average, more than 20 years after the onset of habitual seizures.^4^ A national analysis published a decade after the practice parameter found that surgical volumes in the United States had not risen despite the new evidence and guidelines.^5^

This review revisits the problem 25 years later, We are deliberately U.S.‐centric in our treatment of epidemiology, trends, and disparities, where the data are more comprehensive, while devoting a dedicated section to the international and global picture. We summarize the scope and burden of DRE, the evidence and guideline foundation for surgery, and the magnitude, patterns, and causes of underutilization, with separate attention to children and to health disparities. We then describe genuine progress, especially the expansion of surgical candidacy through minimally invasive monitoring and treatment and the broadening of indications to generalized epilepsies. Finally, we temper enthusiasm with a caution about preserving curative resection, and we close with a call to action.

## 2 THE SCOPE AND BURDEN OF DRUG‐RESISTANT EPILEPSY

Epilepsy is among the most common serious neurological disorders. Throughout this review we follow current ILAE terminology for seizures and the epilepsies.^6^, ^7^ In 2015, an estimated 3.4 million people in the United States (3.0 million adults and 470 000 children), or roughly 1.2% of the population, had active epilepsy.^8^ Across populations, approximately one‐third of patients do not attain lasting seizure control with medication. In the seminal prospective Glasgow cohort, Kwan and Brodie observed that despite the introduction of multiple new agents, a stable minority of patients remained uncontrolled, and that failure to respond to the first one or two well‐chosen drugs strongly predicted ongoing seizures.^9^ This proportion has proved stubbornly fixed: even after the licensing of more than 20 new ASMs over three decades, up to 30%–40% of patients have DRE.^10^ Of note, intractability is not always evident at onset; in a prospective childhood cohort, intractability frequently emerged only after an initial quiescent period, sometimes years into the disease, a pattern that helps explain why surgically remediable epilepsies are so often referred late.^11^

The burden of uncontrolled epilepsy is severe and frequently underappreciated relative to the risks of intervention. DRE carries substantial excess mortality relative to the general population, driven by sudden unexpected death in epilepsy (SUDEP), accidents, drowning, suicide, and status epilepticus, and is associated with cognitive decline, psychiatric comorbidity, injuries, social stigma, loss of driving privileges, unemployment, and impaired quality of life.^4^, ^12^ The mortality burden is tightly linked to seizure control: in surgical cohorts, patients rendered seizure‐free experience mortality rates approaching those of the age‐ and sex‐matched general population, whereas those with persistent seizures carry standardized mortality ratios on the order of 5, with much of the excess attributable to SUDEP.^13^, ^14^ Crucially, these risks of ongoing seizures typically exceed the risks of diagnostic evaluation and surgical treatment, a calculus that argues for earlier rather than later referral.^12^ Against this backdrop, the persistent reluctance to pursue surgical evaluation is not benign watchful waiting but an accrual of avoidable harm.

## 3 THE EVIDENCE BASE AND GUIDELINE FOUNDATION FOR SURGERY

The case for surgery in appropriately selected patients rests on Class I evidence. The Wiebe trial established superiority of resection over continued ASMs for temporal lobe epilepsy.^1^ The Early Randomized Surgical Epilepsy Trial (ERSET) found that 11 of 15 patients randomized to early surgery were seizure‐free in the second year of follow‐up vs none of 23 in the medical group, supporting surgery soon after, rather than decades following, the recognition of pharmacoresistance.^15^ In children and adolescents, a subsequent RCT demonstrated freedom from seizures in 77% of surgically treated patients vs 7% with continued medical therapy at 12 months.^16^ These three trials together constitute the randomized evidentiary backbone of the field across the age spectrum (Table 1).

**TABLE 1:** Randomized controlled trials demonstrating the efficacy of epilepsy surgery.

Trial (year)	Country	Population	Comparison	Primary result
Wiebe et al. (2001)^1^	Canada	Adults, drug‐resistant temporal lobe epilepsy (*n* = 80)	Anteromesial temporal resection vs continued ASMs (1 year)	58% vs 8% free of seizures impairing awareness at 1 year (*p* < .001)
ERSET—Engel et al. (2012)^15^	United States	Adults/adolescents, newly intractable mesial TLE (*n* = 38)	Early anteromesial temporal resection vs continued ASMs (2 years)	11/15 (surgical) vs 0/23 (medical) seizure‐free in year 2 (*p* < .001)
Dwivedi et al. (2017)^16^	India	Children/adolescents ≤18 years, DRE (*n* = 116)	Surgery appropriate to etiology + medical therapy vs medical therapy alone (12 mo)	77% vs 7% seizure‐free at 12 mo (*p* < .001)

Abbreviations: ASM, anti‐seizure medication; DRE, drug‐resistant epilepsy; ERSET, early randomized surgical epilepsy trial; mo, months; TLE, temporal lobe epilepsy.

Observational evidence reinforces and extends these findings. Systematic reviews and large cohorts report long‐term seizure freedom in roughly two‐thirds of patients after temporal lobe resection, with lower but meaningful rates after extratemporal and neocortical resections and after hemispheric surgery.^17^, ^18^, ^19^ A long‐term prospective cohort found that about half of adults remained free of disabling seizures a decade after surgery, with quality of life improving most in those rendered seizure‐free.^19^ A contemporary review summarized median seizure‐freedom rates near 62% and a low perioperative mortality of 0.1%–0.5%.^20^ Two predictors of seizure freedom recur across pathologies and are, importantly, modifiable: earlier operative intervention and, when a discrete lesion is present, its gross‐total resection.^21^ Cognitive outcomes warrant balanced counseling rather than reflexive caution: although many patients are cognitively stable or improved after surgery—especially once seizures cease—dominant‐hemisphere temporal resection can impair verbal memory, a risk that argues for careful neuropsychological selection and for earlier, more tailored surgery before cumulative deficits accrue.^22^

This evidence is mirrored in formal recommendations. Beyond the 2003 AAN/AES/AANS practice parameter,^2^ the ILAE definition of DRE provided an unambiguous, actionable referral trigger,^3^ and the National Association of Epilepsy Centers (NAEC) codified the multidisciplinary services that specialized (Levels 3 and 4) centers should provide.^23^

More recently, the ILAE Surgical Therapies Commission issued expert consensus recommendations on the timing of referral, advising that every patient with drug‐resistant epilepsy up to 70 years of age be referred for surgical evaluation as soon as drug resistance is established—regardless of epilepsy duration, seizure type, or epilepsy syndrome, including the epileptic encephalopathies.^24^ Notably, these recommendations reach beyond drug resistance itself: referral should also be considered for patients, both adults and children, who are seizure‐free on one or two ASMs but harbor a brain lesion in non‐eloquent cortex, based on the rationale that early surgery may be curative and spare years of disability.^24^ As has been argued, whether a lesion's location is genuinely safe to resect is a judgment best made at a specialized center; uncertainty about operability should prompt referral rather than serve as a reason to withhold it.^25^

The guideline architecture, in short, has been in place for the better part of two decades. The problem has never been the absence of evidence or recommendations; it has been their implementation.

## 4 THE MAGNITUDE AND PATTERNS OF UNDERUTILIZATION

If guidelines drove practice, surgical volumes should have risen after 2003. They did not. In a population‐based analysis of U.S. hospitalizations for medically refractory focal epilepsy from 1990 to 2008, the number of resective surgeries did not increase despite rising epilepsy hospitalizations, producing an overall decline in surgery rates even after publication of Class I evidence and the practice parameter.^5^ The same analysis documented a troubling structural shift: a relative migration of epilepsy admissions away from the highest‐volume centers, where surgery is most likely to be performed well, toward lower‐volume hospitals that are less likely to operate.^5^ A single‐center study of referral patterns reached a similarly sobering conclusion: comparing the pre‐guideline era with 2005–2008, there was no statistically significant shortening in the interval from onset of habitual seizures to surgical referral, which remained on the order of 17–18 years^26^ (Table 2).

**TABLE 2:** Representative studies documenting underutilization, referral delay, and utilization trends.

Study (year)	Setting/data source	Key finding
Berg et al. (2006)^11^	Prospective pediatric cohort	Intractability often emerges after an initial quiescent period, helping explain late referral
Haneef et al. (2010)^26^	Single U.S. center, pre‐ vs post‐guideline	No significant shortening of onset‐to‐referral interval (~17–18 years) after the practice parameter
Englot et al. (2012)^5^	U.S. Nationwide Inpatient Sample, 1990–2008	Resective surgery rates did not increase despite Class I evidence and guidelines; disparities by race and insurance
Erba et al. (2012)^27^	Survey of practicing neurologists	Wide variability in attitudes; most respondents diverged from expert opinion on timing and indications
Pestana Knight et al. (2015)^28^	Kids' Inpatient Database, 1997–2009	Pediatric surgery rose (0.85 → 1.44 per 1000) but remained <35% of expected; disparities persisted
Cloppenborg et al. (2016)^29^	German epilepsy center, 1990–2013	Presurgical evaluations rose while resections stayed flat; more complex, non‐lesional case‐mix
Engel (2016)^4^	Wartenberg Lecture (review)	<1% of DRE patients referred; average >20 years from onset to referral
Buttle et al. (2022)^30^	North American survey, pediatric neurologists	Only ~73.5% would refer after two failed ASMs; reluctance to refer generalized EEG patients
Sharma et al. (2025)^31^	U.S. Nationwide Inpatient Sample, 1998–2018	Laser ablation use rose ~6‐fold (2012–2018) as VNS declined; together reach <1% of refractory cases

Abbreviations: ASM, anti‐seizure medication; DRE, drug‐resistant epilepsy; EEG, electroencephalography; VNS, vagus nerve stimulation.

These trends frame the now widely cited estimate that fewer than 1% of patients with DRE are referred for surgical evaluation, and that those who are referred reach a center an average of more than 20 years after seizure onset.^4^, ^32^ Underutilization is not simply a function of too few operations; it reflects a failure of the referral pipeline upstream of the operating room. Many excellent surgical candidates are never recognized as such by general neurologists, and a recurrent misconception is that comprehensive epilepsy centers offer only surgery, when in fact they provide diagnostic re‐evaluation, identification of pseudoresistance, and a spectrum of treatment options.^4^, ^32^, ^33^ The appropriate advocacy, as has been argued, is therefore not narrowly “referral for surgery” but referral of every patient with DRE to a full‐service epilepsy center.^32^, ^33^

## 5 WHY SURGERY REMAINS UNDERUSED: CONTRIBUTING FACTORS AND DISPARITIES

The drivers of underutilization are multifactorial and operate at the level of providers, patients, and systems. Among providers, surveys reveal wide variability in attitudes and substantial divergence from expert opinion. In a survey of practicing neurologists, respondents were markedly more conservative than academic leaders about how long to pursue pharmacotherapy and about the indications for and outcomes of surgery, and attitudes correlated with the number of patients a physician actually referred.^27^ Knowledge gaps, therapeutic inertia, overestimation of surgical risk, and underestimation of the risks of continued seizures all contribute.^27^, ^34^ A particularly consequential misconception is the framing of epilepsy surgery as a merely “palliative” measure of last resort rather than a potentially curative, disease‐modifying treatment; when surgery is viewed as palliation, referral is too readily deferred, while ostensibly palliative pharmacologic options are pursued, to the patient's detriment.^24^, ^35^ Patient‐level barriers include fear of brain surgery, misperceptions about candidacy and outcomes, and concerns about cost and time, whereas system‐level barriers include fragmented care pathways, the logistical burden of multi‐day evaluations, transportation and reimbursement obstacles, and limited access to specialized centers.^34^

Underutilization is not distributed equitably. The national trend analysis found that White patients were more likely to undergo lobectomy than racial minorities, and privately insured patients more likely than those covered by Medicaid or Medicare.^5^ A systematic review by the North American Commission of the ILAE documented disparities in access to surgery by race and ethnicity, alongside broader socioeconomic gradients in epilepsy outcomes.^36^ A dedicated population‐based analysis spanning 16 years found that African American patients were significantly less likely than White patients to undergo anterior temporal lobectomy for temporal lobe epilepsy (odds ratio 0.56), whereas privately insured patients were more likely to be operated upon (odds ratio 1.85)—disparities that remained essentially unchanged over time.^37^ The same fault lines appear in children: those who reach surgery are disproportionately White, privately insured, and from higher‐income, more‐educated families, whereas non‐White and publicly insured children face longer delays to referral and lower rates of surgery.^38^, ^39^ These inequities compound the general problem: the patients least likely to be referred are often those already disadvantaged in access to specialty care. Strategies to overcome these barriers—educational interventions for providers, decision‐support tools embedded in the electronic record, individualized risk–benefit communication for patients, telemedicine, and policy changes addressing affordability and access—have been articulated but remain unevenly implemented.^34^

## 6 PEDIATRIC EPILEPSY SURGERY: A DISTINCT UNDERUTILIZATION PROBLEM

The case for timely surgery is, if anything, more compelling in children, where ongoing seizures and the cumulative effects of ASMs occur during critical windows of neurodevelopment and where surgery can be not only seizure‐controlling but developmentally protective. A pediatric RCT demonstrated dramatic benefit, with 77% of operated children seizure‐free at 1 year.^16^ The ILAE Subcommission for Pediatric Epilepsy Surgery has provided criteria for the referral and evaluation of children, emphasizing earlier consideration and dedicated pediatric resources.^40^ Narrative syntheses underscore both the strength of the evidence and the persistence of underutilization in children.^41^

Pediatric trends offer a partial counterpoint to the adult picture. Using the Kids' Inpatient Database, Pestana Knight and colleagues found that pediatric epilepsy surgery rates rose significantly, from 0.85 to 1.44 surgeries per 1000 children with epilepsy between 1997 and 2009—an encouraging increase, yet one that still reached fewer than 35% of the children expected to benefit, and that again revealed disparities, with lower rates among Black children and those with public insurance.^28^ Referral behavior remains a bottleneck: in a North American survey of pediatric neurologists, only about three‐quarters reported they would refer after two failed medications, and many indicated they would not refer patients with generalized electroencephalographic findings for surgical evaluation—a misconception given the expanding role of surgery and neuromodulation in generalized and multifocal epilepsies.^30^ As in adults, knowledge gaps and family perceptions are modifiable targets for improving utilization.^30^, ^34^ Contemporary practice patterns in children are now being mapped by the ILAE Pediatric Epilepsy Surgery Taskforce. A global survey of invasive presurgical evaluation across 61 programs in 29 countries found that stereo‐electroencephalography (SEEG) has become the dominant invasive technique and that roughly one‐third of invasively studied children have MRI‐negative epilepsy. That survey is one of three forthcoming Taskforce reports, examining referral patterns, invasive EEG, and resection; together they underscore the rising complexity of pediatric surgical candidates.^42^

A particularly compelling rationale for early pediatric surgery is its effect on the developing brain. In a large single‐center cohort of 500 operated children, neuropsychological function—IQ, working memory, processing speed, and academic attainment—declined steadily in the years before surgery, with roughly one‐third to two‐fifths of children losing 10 or more points. Although scores did not change on average across the whole group after surgery, children rendered seizure‐free showed postoperative gains that continued over long‐term follow‐up, and those able to discontinue ASM improved further still.^43^ By achieving seizure freedom and enabling medication withdrawal, surgery may thus not merely halt but improve the cognitive trajectory of childhood drug‐resistant epilepsy—an argument for referral as early as possible after diagnosis.^43^, ^44^ Consistent with this, surgery before the age of 5 years has been associated with preserved or improved developmental and intellectual outcomes.^45^ The developmental stakes of delay in children are therefore especially high.

## 7 THE INTERNATIONAL AND GLOBAL PICTURE

Although this review centers on the United States, underutilization is a worldwide phenomenon, and its character differs across health systems. In high‐income settings outside the United States, surgical volumes have also stagnated or declined, even as presurgical evaluation has expanded. A large German center reported that, despite steadily increasing presurgical evaluations, the number of resective operations remained essentially flat, with declining numbers of “classic” surgically remediable cases such as mesial temporal sclerosis, a rising proportion of non‐lesional and more complex candidates requiring intracranial recordings, and a growing rate of informed patient choice against surgery.^29^ These observations suggest that, in mature programs, part of the apparent plateau reflects more complex case‐mix and better‐informed shared decision‐making rather than referral failure alone.

The starkest disparities, however, are global. A systematic review and meta‐analysis estimated that roughly 10 million people worldwide are current candidates for epilepsy surgery, with ~1.4 million new surgically treatable cases arising each year, the burden falling disproportionately on low‐ and middle‐income countries (LMICs) with the fewest resources.^46^ Epilepsy features prominently among neurosurgical conditions in global surgery analyses, which estimate that millions of essential neurosurgical operations go unmet annually, concentrated in regions such as sub‐Saharan Africa and Southeast Asia.^47^ Country‐level descriptions illustrate the mechanisms: in the Philippines, for example, a large treatment gap reflects scarce specialists, limited access to even basic ASMs, out‐of‐pocket financing, and underutilization of surgery.^48^ In LMICs, the limiting factors are predominantly infrastructural—too few epileptologists, neurosurgeons, neurophysiologists, and imaging resources—whereas in high‐income systems the barriers are more often referral behavior, misconceptions, and case complexity. Both contexts, however, share the same end result: most people who could benefit from epilepsy surgery never receive it. Yet the gap is closable. Demonstration programs suggest that high‐quality epilepsy surgery is feasible even in resource‐limited settings, and coordinated ILAE efforts to expand surgical training and build capacity in underserved regions point toward scalable solutions rather than an immutable deficit.^49^ The evolution of technique has likewise been international rather than uniquely North American: SEEG arose from the European (Talairach–Bancaud) tradition before its worldwide adoption, minimally invasive ablative approaches such as SEEG‐guided radiofrequency thermocoagulation have matured in European and Chinese centers,^50^ and volume‐based thermocoagulation techniques have advanced in Japan,^51^ even as access to any surgical care remains the dominant challenge in low‐ and middle‐income settings.

## 8 SIGNS OF PROGRESS

Notwithstanding the persistent gap, there are real—if still insufficient—grounds for optimism; the central caution of this section is that expanding capability must not be mistaken for closing the gap (Figure 1). The growth and formalization of specialized epilepsy centers, with accreditation standards defining the multidisciplinary services expected of Levels 3 and 4 programs, has expanded the infrastructure for comprehensive evaluation.^23^ Pediatric surgical utilization has measurably increased, even if it remains insufficient.^28^ In mature programs internationally, the expansion of presurgical evaluation—even where it does not always culminate in resection—reflects broader engagement with surgically oriented care and more nuanced, individualized decision‐making.^29^ National data also capture a clear shift in practice: between 2012 and 2018, inpatient use of laser interstitial thermal therapy (LITT) for refractory epilepsy in the United States rose roughly six‐fold (from 0.69 to 4.43 per 1000 cases) even as vagus nerve stimulation use declined.^31^ Yet the same data temper optimism—minimally invasive procedures still reach only a tiny fraction of eligible patients and are concentrated at academic centers and among the privately insured, echoing the disparities long seen for open resection.^31^, ^52^

**FIGURE 1:**
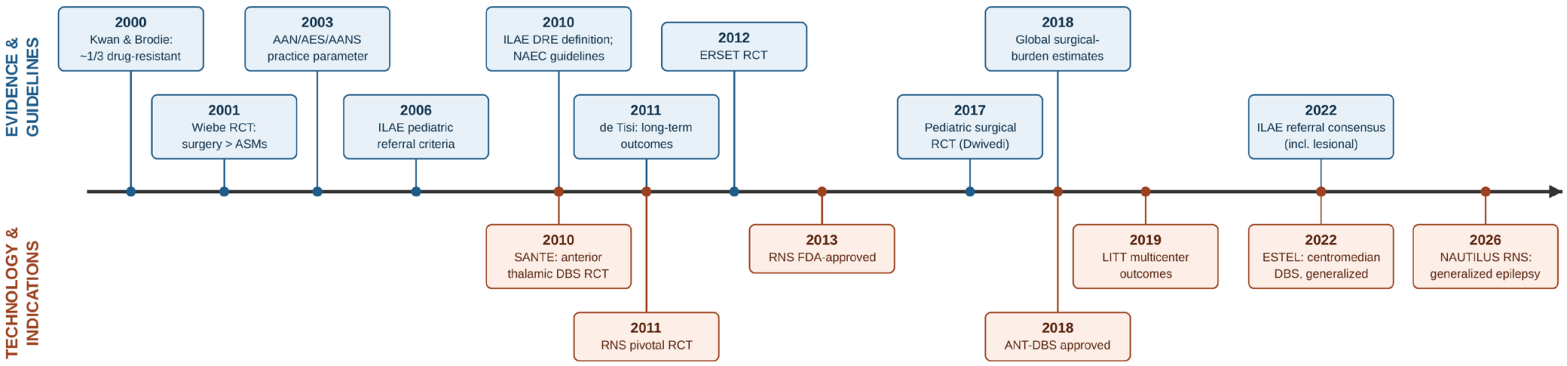
Twenty‐five years of evidence, guidelines, and technological expansion in epilepsy surgery. A timeline of selected milestones from 2000 to the present, including the operational identification of drug‐resistant epilepsy,^9^ the first surgical RCT,^1^ the AAN/AES/AANS practice parameter,^2^ pediatric referral criteria,^40^ the ILAE definition of drug‐resistant epilepsy,^3^ the SANTE trial,^53^ the NAEC revised center guidelines,^23^ the RNS pivotal trial59 and long‐term resective outcome data,^19^ the ERSET trial15 and the national trend analysis showing no increase in surgery,^5^ the pediatric surgical RCT,^16^ global surgical‐burden estimates,^46^, ^47^ the ESTEL trial of centromedian thalamic stimulation for Lennox‐Gastaut syndrome,^54^ and the NAUTILUS trial of responsive centromedian thalamic stimulation for idiopathic generalized epilepsy.^55^ AAN, American Academy of Neurology; AANS, American Association of Neurological Surgeons; AES, American Epilepsy Society; and RCT, randomized controlled trial. The figure juxtaposes the accumulating evidence and guideline base (upper track) against the parallel expansion of minimally invasive monitoring and treatment and broadening indications (lower track), underscoring that growth in capability has not yet been matched by growth in utilization. AAN, American Academy of Neurology; AANS, American Association of Neurological Surgeons; AES, American Epilepsy Society; and RCT, randomized controlled trial.

The most consequential progress, however, is technological and conceptual. The population of patients who can be offered surgical treatment has expanded substantially, and the perceived threshold for intervention has fallen, because of minimally invasive approaches to both seizure localization and treatment, and because of the broadening of indications to epilepsies once considered inoperable. These developments are the subject of the next two sections—although, as argued below, expanded options must not be conflated with expanded delivery, or allowed to displace curative resection where it offers the best chance of cure.

## 9 EXPANDING CANDIDACY: MINIMALLY INVASIVE MONITORING AND TREATMENT

### 9.1 Minimally invasive monitoring

SEEG—the stereotactic placement of depth electrodes through small drill holes rather than via craniotomy for subdural grids—has become a mainstay of invasive evaluation in many centers, enabling three‐dimensional sampling of deep, bilateral, and widely distributed networks with lower morbidity than open approaches. SEEG has extended invasive evaluation to patients who previously would have been deemed too complex or too high‐risk to localize, including those with non‐lesional, bilateral, or multifocal epilepsy. In a large bilateral SEEG cohort, a strong preimplantation hypothesis predicted both proceeding to resection and subsequent seizure freedom, illustrating how SEEG can rationally select and stratify difficult candidates.^56^ It should be noted, however, that philosophies of invasive evaluation differ internationally. In many European programs, lateralization of the epileptogenic zone is expected to be largely established during non‐invasive evaluation, so bilateral implantation accounts for a comparatively small proportion of investigations. Other centers employ bilateral SEEG more liberally to adjudicate competing hypotheses. These are distinct, coexisting traditions rather than a single universal standard.

### 9.2 Minimally invasive treatment

Magnetic resonance (MR)–guided LITT delivers stereotactic thermal ablation of an epileptogenic target through a single small cranial opening, offering shorter hospitalization and recovery than open resection. In a multicenter study of 234 patients undergoing LITT for mesial temporal lobe epilepsy, 58% achieved favorable (Engel Class I) outcomes at 1 and 2 years, with outcomes dependent on adequate ablation of mesial structures.^57^ Refinements such as computer‐assisted and automated trajectory planning aim to improve both safety and ablation adequacy.^58^ Prospective multicenter evaluation of stereotactic laser ablation has further advanced the evidence base for this approach.^59^ LITT exemplifies how a less invasive option can lower the barrier to accepting surgical treatment for patients who decline or are poor candidates for open resection. Beyond laser ablation, other minimally invasive ablative techniques have become well established outside North America. SEEG‐guided radiofrequency thermocoagulation (RF‐TC), which uses the implanted depth electrodes themselves to create small thermolesions, has become an important therapeutic—and prognostic—option in several European and Chinese epilepsy surgery programs, providing seizure relief in a meaningful subset of patients and helping to inform subsequent resection.^50^ In Japan, volume‐based stereotactic RF‐TC has been developed and refined, most extensively for hypothalamic hamartoma, where it can achieve high rates of freedom from gelastic seizures.^51^ Together with LITT, these techniques reflect a broader international shift toward stereotactic ablation as an alternative or complement to open resection.

### 9.3 Advances in conventional surgery

Progress over the past 25 years has not been confined to minimally invasive methods; open resective and disconnective surgery has itself matured. Multimodal structural and functional imaging, electroencephalography–MR imaging and magnetoencephalographic source localization, and refined electrical and functional mapping have sharpened the delineation of the epileptogenic zone and eloquent cortex; frameless neuronavigation and intraoperative imaging have improved the precision and safety of resection; disconnective procedures such as functional hemispherotomy and corpus callosotomy have reduced the morbidity of large interventions; and the integration of neuropathological and genetic knowledge—particularly for focal cortical dysplasia and developmental tumors—has increasingly informed surgical decision‐making and prognosis.^33^, ^60^ These conventional advances complement, rather than compete with, the minimally invasive expansion described above.

### 9.4 Neuromodulation

For patients who are not candidates for resection or ablation—because the seizure focus is unresectable, eloquent, multifocal, or non‐localizable—neuromodulation has transformed the therapeutic landscape. Vagus nerve stimulation (VNS), the longest‐established option, produces meaningful seizure reduction with response rates that increase over time, although complete seizure freedom is uncommon.^61^ Brain‐responsive neurostimulation (RNS), which detects and responds to epileptiform activity at one or two seizure foci, demonstrated efficacy in its pivotal RCT (a 37.9% reduction in seizures during the blinded phase vs 17.3% with sham)^62^ and showed progressive, durable benefit in long‐term follow‐up, with a median seizure reduction of roughly 75% at 9 years.^63^ Deep brain stimulation of the anterior nucleus of the thalamus (ANT‐DBS), validated in the SANTE (Stimulation of the Anterior Nucleus of Thalamus for Epilepsy) trail, produced a 56% median reduction in seizures at 2 years with continued improvement thereafter.^53^ Comparative and review syntheses note that, although complete seizure freedom is achieved in only a minority of patients, these neuromodulatory therapies substantially reduce seizure burden and improve quality of life; they are therefore better characterized as disease‐modifying than as merely palliative. Indeed, the pejorative framing of neuromodulation—and of epilepsy surgery more broadly—as palliation understates its benefit and may itself discourage timely referral, and is best avoided.^35^, ^64^ Taken together, SEEG, LITT, and neuromodulation have widened candidacy at both ends: enabling localization in patients once deemed unlocalizable, and offering treatment to patients once deemed inoperable^33^ (Table 3).

**TABLE 3:** Minimally invasive and neuromodulatory approaches expanding surgical candidacy.

Modality	Role	Key evidence	Seizure‐freedom potential
Stereo‐electroencephalography (SEEG)	Minimally invasive monitoring/localization	Bilateral SEEG cohort: strong preimplantation hypothesis predicts resection and seizure freedom^56^	Diagnostic (enables resection in complex cases)
Laser interstitial thermal therapy (LITT)	Minimally invasive ablation (e.g., mesial temporal)	Multicenter cohort (*n* = 234): 58% Engel Class I at 1–2 years^57^; prospective multicenter study^59^; trajectory optimization^58^	Potentially curative; no RCT vs resection
Vagus nerve stimulation (VNS)	Extracranial neuromodulation	Registry/systematic review: responders increase over time; ~8% seizure‐free^61^	Predominantly disease‐modifying
Responsive neurostimulation (RNS)	Closed‐loop neuromodulation at 1–2 foci	Pivotal RCT: −37.9% vs −17.3% sham^62^; ~75% median reduction at 9 years^63^	Predominantly disease‐modifying; rarely seizure‐free
Anterior nucleus thalamic DBS (ANT‐DBS)	Neuromodulation for focal epilepsy	SANTE RCT: 56% median reduction at 2 years^53^	Predominantly disease‐modifying
Centromedian thalamic neuromodulation (CM‐DBS/CM‐RNS)	Neuromodulation for generalized/network epilepsy	ESTEL RCT (CM‐DBS) in Lennox–Gastaut syndrome^54^, ^65^; NAUTILUS RCT (CM‐RNS) in idiopathic generalized epilepsy^55^—NAUTILUS: prespecified primary efficacy endpoint not met; post hoc analysis 61% vs 49%; open‐label median reduction 76.8% at 18 months	Predominantly disease‐modifying; investigational for generalized epilepsy (CM‐RNS not FDA‐approved)

Abbreviations: ANT‐DBS, anterior nucleus of the thalamus deep brain stimulation; CM‐DBS, centromedian deep brain stimulation; CM‐RNS, centromedian responsive neurostimulation; DBS, deep brain stimulation; ESTEL, Electrical Stimulation of the Thalamus for Epilepsy of Lennox‐Gastaut phenotype; FDA, US Food and Drug Administration; LITT, laser interstitial thermal therapy; RCT, randomized controlled trial; RNS, responsive neurostimulation; SANTE, Stimulation of the Anterior Nucleus of Thalamus for Epilepsy; SEEG, stereo‐electroencephalography; VNS, vagus nerve stimulation.

## 10 NEW INDICATIONS: NEUROMODULATION FOR GENERALIZED AND MULTIFOCAL EPILEPSIES

Perhaps the clearest expansion of indications is the extension of surgical therapy beyond focal epilepsy into generalized and network epilepsies historically regarded as non‐surgical. Thalamic neuromodulation is central to this shift. In the NAUTILUS pivotal randomized trial—the first controlled neuromodulation study in idiopathic generalized epilepsy—responsive stimulation of the centromedian thalamus was evaluated in patients with drug‐resistant generalized tonic–clonic seizures; although the prespecified primary efficacy endpoint was not met, post hoc analysis favored active stimulation (61% vs 49% reduction in generalized tonic–clonic seizures), and open‐label follow‐up showed a 76.8% median seizure reduction at 18 months.^55^ In the ESTEL (Electrical Stimulation of the Thalamus for Epilepsy of Lennox‐Gastaut phenotype) randomized trial, DBS of the centromedian nucleus of the thalamus in young adults with Lennox–Gastaut syndrome—a severe, typically generalized epileptic encephalopathy—reduced seizures, with half of stimulated participants achieving at least a 50% reduction in diary‐recorded seizures and a clearer effect on electrographic seizures.^54^ Functional and caregiver‐reported measures suggested concomitant reductions in epilepsy severity and disability.^65^ More broadly, the thalamus has emerged as a rational target for network‐level modulation in generalized and multifocal epilepsies, and the role of responsive and deep brain stimulation continues to widen as evidence accrues.^64^ These developments matter for utilization because they directly counter one of the most common reasons patients—especially children with generalized electroencephalographic findings—are never referred: the belief that nothing surgical can be offered.^30^

## 11 A NOTE OF CAUTION: PRESERVING RESECTION IN THE MINIMALLY INVASIVE ERA

The expansion of minimally invasive monitoring, ablation, and neuromodulation is, on balance, a major advance, and it has almost certainly broadened access to surgically oriented care. Yet it warrants a counterbalancing caution. Resective (and, in selected cases, disconnective) surgery remains the only potentially curative therapy for drug‐resistant epilepsy that is supported by Class I randomized evidence—the temporal lobe trials of Wiebe and ERSET in adults^1^, ^15^ and the pediatric trial demonstrating benefit across surgical procedures in children.^16^ By contrast, laser ablation, despite encouraging cohort data and prospective multicenter study, has not been tested against resection or medical therapy in an RCT,^57^, ^59^ and neuromodulation, although RCT‐validated, is fundamentally disease‐modifying, with complete seizure freedom achieved in only a small proportion of patients.^53^, ^62^, ^63^, ^64^ Long‐term seizure freedom after temporal lobe resection remains higher than that reported for less invasive alternatives.^18^

There is, therefore, a legitimate concern that a wholesale shift toward minimally invasive options—driven by understandable patient and physician preferences for smaller procedures—could, if applied indiscriminately, trade the prospect of cure for the convenience of a smaller intervention. The appropriate response is not to resist innovation but to integrate it. Every patient with DRE deserves evaluation at a comprehensive center where the full menu of options, including curative resection, is presented honestly, and where the choice among resection, ablation, and neuromodulation is matched to the individual's epilepsy, goals, and the realistic probability of seizure freedom each approach offers.

A second, less obvious risk concerns the surgical workforce. Minimally invasive procedures—laser ablation, stereotactic electrode implantation, and device placement—are, from a technical standpoint, more readily accomplished with less open operative experience than the complex resections and disconnections that remain necessary for many patients. Minimizing invasiveness is itself a major advance, but as case volumes migrate toward these less demanding procedures, an as‐yet‐unproven but plausible consequence could be a shrinking pool of surgeons who maintain competence in technically demanding open epilepsy surgery. Preserving that expertise—through deliberate training, adequate case volumes, and regionalization of complex cases at high‐volume centers—is essential if the field is to retain its most effective, potentially curative tools.^31^, ^49^, ^52^

## 12 THE ECONOMIC CASE FOR TIMELY REFERRAL

The argument for referral is not only clinical but economic. People with drug‐resistant epilepsy constitute roughly 40% of those with epilepsy yet account for ~80% of epilepsy‐related expenditure, the bulk of it driven by uncontrolled seizures, recurrent hospitalizations, emergency care, and chronic medication use.^4^ Against this backdrop, surgery is not a costly luxury but, for appropriate candidates, a cost‐saving investment. In a U.S. lifetime decision‐analytic model, resective surgery for drug‐resistant temporal lobe epilepsy was both more effective and less expensive than continued medical management (16.6 vs 13.6 quality‐adjusted life‐years; $328 000 vs $423 000), and referral for surgical evaluation was cost‐effective even when the probability of ultimately proving to be a surgical candidate was as low as 5%; from a societal perspective, surgery became cost‐saving within roughly 3 years.^66^ Empirical cost data concur: among patients who become seizure‐free after temporal lobe surgery, total health care costs fall by about one‐third within 2 years—chiefly through reduced ASM and inpatient use—whereas costs remain flat in those whose seizures persist, with or without surgery.^67^ The economic logic thus reinforces the clinical one: delaying or withholding referral does not save money so much as defer and compound it. Payers and health systems have a direct financial stake in earlier referral, and cost‐effectiveness should be understood as an argument for, not against, timely evaluation at a comprehensive center.^66^

## 13 A CALL TO ACTION: RECOMMENDATIONS FOR THE FUTURE

Approximately 25 years after Wiebe and the practice parameter, the central conclusion is unavoidable: epilepsy surgery remains substantially underutilized, and the gap between evidence and practice represents a large, partly remediable burden of avoidable disability and premature death.^4^, ^5^, ^32^ Closing this gap will require coordinated action across several fronts.

First, the referral trigger must be made operational and routine. Every patient who continues to have seizures after two appropriately chosen and tolerated ASMs meets the definition of DRE and should be referred to a comprehensive epilepsy center—not for surgery per se, but for comprehensive evaluation of all options.^3^, ^32^ Contemporary ILAE consensus goes further, recommending referral even for select patients who are seizure‐free on one or two medications but harbor a resectable lesion, with the question of operability decided at the specialized center rather than by the referring provider.^24^, ^25^ Embedding this trigger in electronic health records, quality metrics, and primary and general neurology workflows can reduce reliance on individual judgment.^34^ In Engel's words, “all patients with medication‐resistant epilepsy … who continue to be compromised by seizures deserve a timely consultation at a full‐service epilepsy center,” because “early referral provides the best opportunity to avoid irreversible psychological and social problems, a lifetime of disability, and premature death.”^4^

Second, provider education must directly target the misconceptions that drive underutilization: that centers offer only surgery, that surgery is a last resort, that generalized or non‐lesional epilepsy precludes intervention, and that the risks of surgery outweigh those of ongoing seizures.^27^, ^30^, ^33^ Reframing comprehensive centers as offering diagnosis, pseudoresistance identification, and a spectrum of therapies—including the newer minimally invasive and neuromodulatory options—may lower the perceived stakes of referral. Education, moreover, cannot stop at neurologists: primary care physicians and emergency providers are often the first—and sometimes only—clinicians a patient sees; patients and families need accurate information to seek and accept referral; payers shape access through coverage and reimbursement; and public‐awareness campaigns can reduce the stigma and misconceptions that deter care. Each is a distinct and necessary target.^33^

Third, equity must be explicit. Interventions should be designed and evaluated for their effect on the racial, insurance‐based, and geographic disparities that consistently shape who reaches surgery, with attention to transportation, reimbursement, telemedicine, and the affordability of multi‐day evaluations.^5^, ^34^, ^36^

Fourth, the pediatric pathway deserves particular urgency, given the developmental stakes and the demonstrated, if insufficient, gains already achieved; earlier referral criteria should be widely adopted.^28^, ^30^, ^40^

Fifth, capacity must expand where it is scarcest. Globally, narrowing the surgical treatment gap will require investment in workforce and infrastructure in LMICs, integrated into broader strengthening of surgical and neurological systems.^46^, ^47^, ^48^

Finally, innovation should be harnessed without losing sight of cure. Minimally invasive monitoring and treatment and the broadening of indications to generalized epilepsies should be deployed to widen access, while resection is preserved and offered wherever it provides the best chance of seizure freedom.^20^, ^54^ Continued comparative research—ideally including randomized comparisons of minimally invasive ablation against resection—would help match each therapy to the patients it serves best.^57^, ^60^

## 14 CONCLUSION

The story of epilepsy surgery utilization over the past quarter‐century is one of both peril and progress. The peril is the enduring, well‐documented failure to deliver an effective, sometimes curative therapy to the patients who need it: fewer than 1% referred, decades of delay, and disparities that fall hardest on the already disadvantaged. The progress is genuine and accelerating: minimally invasive monitoring and treatment have expanded who can be offered surgery and lowered its threshold, and neuromodulation has carried surgical therapy into generalized and network epilepsies once thought beyond reach, although it does not replace surgical resection. The task for the next 25 years is to ensure that these advances close the gap rather than merely widen the menu. Every patient with drug‐resistant epilepsy, regardless of age, geography, or means, should be evaluated in time to benefit, and the choice among an expanding array of options should always include the one most likely to render the patient seizure‐free.

## AUTHOR CONTRIBUTIONS

D.J.E. conceived the work, performed the literature review, and wrote, revised, and approved the manuscript.

## FUNDING INFORMATION

This research received no specific grant from any funding agency in the public, commercial, or not‐for‐profit sectors.

## CONFLICT OF INTEREST STATEMENT

The author is an investigator on studies of thalamic neuromodulation, including centromedian stimulation in generalized epilepsy, and has received consulting reimbursement from NeuroPace. He has also received travel and meal support from NeuroPace for educational content.

## ETHICAL PUBLICATION STATEMENT

We confirm that we have read the Journal's position on issues involved in ethical publication and affirm that this report is consistent with those guidelines.

## Data Availability

Data sharing is not applicable to this article, as no new data were created or analyzed.
